# Family Environment and Portuguese Adolescents: Impact on Quality of Life and Well-Being

**DOI:** 10.3390/children9020200

**Published:** 2022-02-03

**Authors:** Fábio Botelho Guedes, Ana Cerqueira, Susana Gaspar, Tania Gaspar, Carmen Moreno, Margarida Gaspar de Matos

**Affiliations:** 1 Institute of Environmental Health (ISAMB), Aventura Social, Faculty of Medicine, University of Lisbon (FMUL), 1649-028 Lisbon, Portugal; cerqueira.apm@gmail.com (A.C.); smsgaspar@gmail.com (S.G.); tania.gaspar.barra@gmail.com (T.G.); margarida.gaspardematos@gmail.com (M.G.d.M.); 2 Faculty of Human Kinetics, University of Lisbon/FMH-UL, 1495-751 Lisbon, Portugal; 3 Lusíada Center for Research in Social Work and Social Intervention (CLISSIS), Lusíada University of Lisbon, 1349-001 Lisbon, Portugal; 4 Atlântica Health School, Atlântica University, 2730-036 Barcarena, Portugal; 5 Department of Developmental and Educational Psychology, University of Seville, 41018 Seville, Spain; mcmoreno@us.es; 6 APPSYci, ISPA—University Institute, 1100-304 Lisbon, Portugal

**Keywords:** adolescents, quality of life, family environment, family relationship, family support

## Abstract

Background: A healthy and supportive family environment leads to more positive results regarding adolescents’ development. The main objective of this study was to explore and analyze the relationship between adolescents’ quality of life (QoL) and their family environment/relationship. Method: The sample was collected as part of the Health Behavior in School-aged Children 2018 study, which included 8215 adolescents, 52.7% female, with a mean age of 14.36 years (*SD* = 2.28). Results: Girls are more involved in family activities (such as family meals), report being treated with fairness by their parents and feel less parental pressure to get good grades. Boys have a higher perception regarding their family affluence, better family relationships and support and better QoL. Having an above-average QoL is significantly related to high family affluence, better communication with both parents, greater involvement in family activities, greater perception of help from parents regarding decision-making, greater perception of being treated with fairness by parents and less pressure from parents to get good grades, as well as a better family relationship/support. Conclusion: It is important to determine the impact that parental divorce/separation or a weak parent–child relationship can have on adolescents. It is also necessary to consider the family relationship and structure when devising strategies and public policies related to the promotion of adolescents’ health and well-being.

## 1. Introduction

The family is one of the main contexts in which the development of children and adolescents takes place. The dynamics that occur in this context are likely to influence their experiences and growth. Evidence indicates that the greater the instability experienced in this context, the worse the developmental outcomes of the adolescents [1,2,3,4,5]. Thus, a more adjusted family function and a closer relationship between parents and children are factors that are associated with a more positive and adjusted development and a higher level of well-being [3,6,7,8].

Family configurations have undergone several changes over time, with an increase in the number of families consisting of divorced or separated parents and several single-parent families [2,9]. These scenarios have repercussions regarding adolescents’ well-being [2,3,4,10,11,12]. Parental separation has a significative impact on the lives of children and adolescents. These events expose them to several changes that require adaptability and that can compromise their development and psychosocial well-being [9,13,14,15].

A healthy family environment includes characteristics related to proximity, concern and support and is reflected in a greater well-being and quality of life and more positive results [16]. Supportive parental relationships also produce more positive results, as opposed to relationships based on rejection, which can lead to the emergence of developmental problems [15]. Thus, the existence of relationships between parents and children based on trust, communication and absence of alienation is associated with adolescents’ well-being [17].

Parent–child relationships are very important with regard to the results obtained by adolescents [17,18,19]. These relationships undergo several changes throughout the developmental process and adolescence has a significant impact on these types of interactions [20,21,22]. Parental monitoring is an important aspect regarding adolescents’ development, as it is a source of support and guidance that can facilitate their journey throughout adolescence [21,23].

The concept of quality of life is related to the perception that individuals have of different aspects of their lives, which include the family and the school environment and relationships with peers [24]. Quality of life can be influenced by several factors and the results in this domain vary according to the gender and age of the adolescents [18,24,25,26,27]. According to Wallander [28], the quality of life of children and adolescents results from a combination of subjective and objective well-being in relation to different areas of their lives, framed in a specific context and culture and bearing in mind universal human rights.

Since the family is one of the privileged environments for the socialization of children and adolescents, their level of participation in this context is likely to affect their well-being. Adolescents who feel that they can communicate their ideas and opinions more openly and who feel involved in family decisions (with a level of participation properly adjusted to their stage of development) tend to obtain better results in terms of well-being [29]. Good communication is also a relevant factor regarding a family environment that promotes development and quality of life [30,31,32].

Another important aspect that can have an impact on adolescents’ quality of life and well-being is related to family meals. These are moments of sharing between family members, contributing to an increase in communication, closer relationships and better family functioning [16,33,34]. The pressure exerted by parents regarding academic performance is also a factor likely to influence the well-being of adolescents [35,36].

Socioeconomic status is a relevant variable with regard to the results obtained by adolescents and can compromise their well-being and quality of life [8,18,23,37,38,39]. Evidence points to the existence of a greater propensity to engage in risk behaviors in families experiencing financial stress situations [40,41].

Finally, the literature shows that boys tend to perceive a higher level of well-being and quality of life when compared to girls [24,27,35,39,42,43]. The same is true for younger adolescents compared to older ones [27,42].

Considering the importance of the family in relation to adolescents’ development, this study aims to analyze the impact that the family environment and relationships have on the perception of quality of life and on the well-being of adolescents.

## 2. Method

This study is based on the Health Behavior in School aged Children/HBSC [44,45], a survey carried out every 4 years, in collaboration with the World Health Organization (WHO), following an international protocol [46]. It has been developed in Portugal since 1998. The data collected aim to study adolescents’ behavior in their life contexts and the influence on their health/well-being.

In Portugal, the HBSC 2018 was approved by the Ethics Committee of the Hospital de S. João do Porto and by MIME (Monitoring Surveys in School Environments). The approval date was 4 January 2018. School groups voluntarily agreed to participate, and informed consent was obtained from the parents or legal guardians of all students. The responses to the questionnaire were obtained online and anonymously. More details on the data collection procedures of the HBSC study in Portugal can be found in Matos and Equipa Aventura Social [45].

### 2.1. Participants

A total of 8215 adolescents were included, of which 52.7% (*N* = 4327) were female, with a mean age of 14.36 years (*SD* = 2.28). The sample includes students from the 6th (30.7%), 8th (33.7%), 10th (20.8%) and 12th (14.8%) school grade and is proportionally distributed over the five Portuguese regions (Norte, Centro, Lisbon and Tagus Valley, Alentejo and Algarve).

### 2.2. Measures and Variables

Taking into account the objective under study, the following variables presented in Table 1 were considered.

## 3. Data Analysis

Data were analyzed using SPSS (Statistical Package for the Social Sciences) version 25 for IOS. Descriptive statistics were performed to characterize the sample. The Chi-Square Test for independent variables was used to analyze the relationship between gender and quality of life and sociodemographic characteristics (age, education, region and FAS), communication with the father and mother, living with both parents in the same house, family meals, being treated with fairness by the parents, parental pressure to get good grades and parental employment. Independent-sample *t*-tests were used to analyze the relationship between quality of life and family relationships and support.

The association between quality of life and significant variables for the adolescents’ family environment was analyzed using a linear regression model, adjusted for age and gender. A significance level of *p* < 0.05 was determined.

## 4. Results

For this study, all students were considered for the analysis of the quality of life related to the family environment. Table 2 presents the characteristics of the population, as well as the analysis of their differences regarding family and gender, where 52.7% (*N* = 4327) are girls. Statistically significant differences were found between gender and school grade, region, family affluence, communication with parents, family meals, parental help to make decisions, parental pressure to have good grades, quality of family relationships, family support and quality of life.

Girls have more difficult communication with their father and mother and feel less help from their parents regarding decision making, when compared to boys. On the other hand, girls have more family meals, feel that they are treated with fairness by their parents and report less parental pressure to get good grades. Boys have a higher perception of family affluence, report a better relationship with the family, greater family support and a better quality of life when compared to girls.

Table 3 presents the bivariate analysis of the differences in quality of life (below or above average) of Portuguese adolescents and the relationship with the variables related to the family environment. Statistically significant differences were found between the quality of life of students and all the other variables under study.

An above-average QoL is statistically and significantly related to being a boy, being younger (8th grade) and being from the northern region. It is also related to high family affluence (including parents being employed), better communication with both parents, living in the same house with both parents, having family meals, having parental help to make decisions (and not having the parents make the decisions for them), being treated with fairness and not being pressured by parents to get good grades. 

Additionally, having an above-average QoL is statistically and significantly associated with having better family relationships and greater family support.

The model presented in Table 4 intends to understand and explain the impact of variables related to the family environment on the quality of life of adolescents. The model includes significant variables in the bivariate analysis (Table 3) adjusted for sex and age, *F*(11,3734) = 211,54; *p* ≤ 0.001, and presents an explanatory value of the variance of 38.3%.

According to this model, the QoL is better explained and has a positive relationship with the adolescents who feel treated with fairness by their parents with the quality of the relationship they have with their family and with the family support provided, as well as with family affluence. On the other hand, communication with parents, the help that parents give to make decisions and parental pressure to have good grades have a negative relationship with the perception of quality of life.

## 5. Discussion

The results present in this study highlight the existence of statistically significant differences between boys and girls. Girls have more difficult communication with their parents, but they are the ones who are more involved in family activities (such as family meals), show a greater perception of fairness in the way they are treated by their parents and less parental pressure regarding school results.

The literature shows gender differences in terms of communication with parents, with boys tending to obtain more positive results than girls regarding communication with their fathers [50,51]. In the same sense, Levin et al. [52] observed that girls have more difficulties in communicating with at least one parent. Elgar et al. [53] obtained similar results but regarding both parents. The results of a study by Xiao et al. [54] allow us to observe that boys have the lowest levels of openness in communication with their parents.

It should be noted that the differences regarding the easiness/difficulty of communicating with parents vary across the various waves of the HBSC study and from country to country [55]. In this way, the differences and inconsistencies in the results obtained by boys and girls in relation to this dimension may be due to the demographic and cultural characteristics of the samples used in the different studies presented in the literature.

A study by Fulkerson et al. [56] showed that girls have more family meals compared to boys. Elgar et al. [53] observed that family dinners are more frequent among girls. On the other hand, a study by Neumark-Sztainer et al. [57] points out that girls tend to report fewer family meals. The authors also found differences between racial groups which indicate that it is important to consider cultural issues in the analysis of these results. A study by Harrison et al. [16] demonstrated that family meals were negatively associated with a variety of risk behaviors (violent behavior, alcohol and substance use) and positively associated with self-esteem and school success. Regular family meals seem to have a more protective effect on girls [16,34].

The literature evidences the existence of differences between boys and girls in terms of academic success. The results of this study regarding girls feeling less parental pressure to obtain good grades may be due to their tendency to present better academic results compared to boys [58,59]. Other variables must be considered when exploring gender differences regarding academic results, namely expectations. Girls tend to have more expectations of continuing their studies for higher education, which may be reflected in the differences found between genders in terms of academic results [58,59,60].

Boys have a greater perception of their family’s socioeconomic status, as well as a greater perception of quality of life, better family relationships and better support. This is in line with the literature, which shows that boys are the ones who perceive a higher level of quality of life and well-being. There is also evidence that the socioeconomic status of the family is an important factor with a direct influence on the well-being and quality of life of adolescents [18,23,24,27,35,37,38,39,42,43].

Adolescents with an above-average quality of life are usually the ones with the greatest family support and involvement. They have greater family stability (they eat more meals as a family and live in the same house as their parents) and a better relationship and communication with their parents. They also feel that they can count on their parents and that they are treated with fairness. On the other hand, a family environment marked by greater instability is a risk factor for adolescents’ development [2].

The literature points to the family configuration as a relevant aspect in terms of the results obtained by adolescents. Those who live in single-parent environments tend to have more impairments in behavioral and socioemotional domains when compared to the ones who live in nuclear families [2]. In the same sense, the adolescents who live with both parents tend to have higher levels of well-being [3,11,18].

The literature also points to the existence of associations between developmental and behavioral problems resulting from frequent changes in their parents’ marital status. This tends to lead to greater family instability [13] and greater inconsistencies in parental behavior [15]. However, it is important to consider other factors associated with the family environment that may underlie these variations regarding adolescents’ well-being [3].

A study by Moore et al. [61] showed that positive family relationships are associated with better developmental results and with a higher level of well-being and quality of life. The communication quality is an aspect that contributes to the relationships between parents and children and, consequently, is an aspect likely to influence adolescents’ development [30,32]. Thus, there is evidence that family activities (e.g., family meals) is an aspect that influences the well-being and quality of life of adolescents [16,33,34].

Jiménez-Iglesias et al. [42] showed that parental affection, carrying out family activities and promoting autonomy are factors that are associated with quality of life. Similarly, a study by Duineveld et al. [62] concluded that the autonomy that parents give to their children is negatively associated with depressive symptoms and positively associated with self-esteem.

Mínguez [18] revealed that the well-being of adolescents is related to factors such as gender, family structure, social relationships (i.e. family, friends and teachers) and safety of the area of residence. Evidence also points towards the fact that the family structure is associated with adolescents’ psychosocial well-being [2,3,4,10,11,12].

This study has some limitations that should be considered, such as the fact that the data are self-reported (there may be bias on the part of the adolescents). It is also a cross-sectional study, which does not allow us to make inferences about causality. In addition, the data only include students from the public education system, and it is not possible to generalize these results to all Portuguese adolescents (i.e., students who dropped out of school or those from the private education system were not included). Nevertheless, it is necessary to bear in mind that the HBSC is a large-scale study, with a very rigorous methodology that is followed by all the countries in the HBSC network. This allows for comparisons to be made with other countries or with data obtained over the several years of study in Portugal.

## 6. Conclusions

The results reveal the importance that the family environment has in the adolescents’ development, as well as in their quality of life and well-being. Family activities and contact with both parents proved to be fundamental for their adjustment and well-being. It is important to determine the impact that parental divorce/separation or a weak parent–child relationship can have on adolescents.

Family relationships and psychosocial factors can influence the parental role and consequently adolescents’ well-being. This reinforces the need to take these variables into account when designing strategies and public policies related to the promotion of adolescents’ health and well-being.

For the harmonious development of adolescents and the consequent impact on their quality of life, it is important to develop and implement intervention programs that can promote family activities (even when parents are separated). These interventions aim to promote time spent in the family, parent–child communication and more positive family relationships. In addition, they also aim to value family diversity and the promotion of socio-emotional skills [45,63,64]. On the other hand, it is necessary to promote and develop greater support for families, regarding the promotion of personal and social skills. This work can be conducted on an individual level (i.e., with each family member) but will have an impact on the overall functioning of the family.

From a public policies point of view, namely in the education, work, leisure and parenting areas, it is important that family quality time is prioritized, with the aim of regulating and optimizing relationships. Furthermore, it is important that families are supported in their problem solving and conflict management strategies, in order to favor the family ethos which is a factor of capital importance for the well-being of all family members, as this study demonstrates.

## Figures and Tables

**Table 1:** Measures and variables under study.

Variables	Measures
Gender	1—Male; 2—Female
School grade	1—6th grade; 2—8th grade; 3—10th grade; 4—12th grade
Region	1—North; 2—Center; 3—Lisbon and Tagus Valley; 4—Alentejo; 5—Algarve
Family affluence	FAS Scale—Family Affluence Scale, with 6 items that reflected the material resources of the family, such as owning a car or individual computer. The FAS score [47,48] was calculated for each adolescent based on the responses to these 6 items, on a scale ranging from 0 to 13 points, with the highest values indicating better financial level.1—Low; 2—Medium; 3—High
Communication with father	1—Easy; 2—Difficult
Communication with mother	1—Easy; 2—Difficult
Living with both parents	1—No; 2—Yes
Family meals	1—No; 2—Yes
Parents help in decision making	1—No; 2—Yes
Parents treat with fairness	1—No; 2—Yes
Pressure from parents to get good grades	1—No; 2—Yes
Father’s employment	1—Employed; 2—Unemployed
Mother’s employment	1—Employed; 2—Unemployed
Quality of family relationship	Scale adapted from Cantril [49], consisting of 11 steps, where the lowest step (0) corresponds to the worst quality of family relationship and the highest step (10) to the best quality of family relationship.
Family support	Scale with 4 items, on a 7-point Likert scale, 1 being very strongly disagree and 7 very strongly agree. Higher values reveal greater family support. α = 95.
Quality of life	Scale with 10 items with scores from 0 to 5. Minimum scores of 5 and maximum score of 50. Higher values reveal a better perception of quality of life. α = 83. The variable was dichotomized taking as reference and mean QoL of young people (Below average/Above average).

**Table 2:** Population characteristics.

	*M* ± *SD* or % (*n*)			*p*
	Total (*N* = 8215)	Boys 47.3% (*N* = 3888)	Girls 52.7% (*N* = 4327)	
Age (years) ^1^	14.36 ± 2.28	14.31 ± 2.28	14.40 ± 2.28	0.068
School grade ^2^				≤0.001
6th grade	30.7 (2520)	* 32.4 (1260)	29.1 (1260)	
8th grade	33.7 (2766)	34.4 (1336)	33.0 (1430)	
10th grade	20.8 (1711)	20.1 (782)	21.5 (929)	
12th grade	14.8 (1218)	13.1 (510)	* 16.4 (708)	
Region ^2^				0.002
North	40.9 (3360)	* 42.8 (1664)	39.2 (1696)	
Centre	16.9 (1390)	16.4 (638)	17.4 (752)	
Lisbon and Tagus Valley	23.5 (1927)	21.8 (847)	* 25.0 (1080)	
Alentejo	9.2 (754)	9.1 (355)	9.2 (399)	
Algarve	9.5 (784)	9.9 (384)	9.2 (400)	
Family affluence ^1^	7.98 ± 2.32	7.98 ± 2.32	7.98 ± 2.32	≤0.001
Low ^2^	26.7 (2112)	23.8 (927)	* 27.4 (1185)	
Medium ^2^	47.3 (3887)	47.2 (1834)	47.4 (2053)	
High ^2^	27.0 (2216)	* 29.0 (1127)	25.2 (1089)	
Communication with father ^2^				≤0.001
Easy	69.7 (4895)	* 77.3 (2590)	62.9 (2305)	
Difficult	30.3 (2123)	22.7 (761)	* 37.2 (1362)	
Communication with mother ^2^				0.013
Easy	85.7 (6419)	* 86.7 (3074)	84.7 (3345)	
Difficult	14.3 (1073)	13.3 (470)	* 15.3 (603)	
Living with both parents ^2^				0.237
No	28.0 (1237)	27.1 (532)	28.7 (705)	
Yes	72.0 (3184)	72.9 (1432)	71.3 (1752)	
Family meals ^2^				≤0.001
No	1.9 (155) 98.1	* 2.5 (95)	1.4 (60)	
Yes	(7797)	97.5 (3672)	* 98.6 (4125)	
Parents help in decision making ^2^				≤0.001
No	15.5 (1188)	14.0 (508)	16.9 (680)	
Yes	84.5 (6460)	* 86.0 (3109)	83.1 (3351)	
Parents treat with fairness ^2^				0.717
No	15.6 (775)	15.8 (358)	15.4 (417)	
Yes	84.4 (4188)	84.2 (1905)	* 84.6 (2283)	
Pressure from parents to get good grades ^2^				≤0.001
No	45.5 (2159)	41.1 (880)	* 49.0 (1279)	
Yes	54.5 (2590)	* 58.9. (1259)	51.0 (1331)	
Father’s employment ^2^				0.681
Employed	94.1 (6655)	94.2 (3167)	93.9 (3488)	
Unemployed	5.9 (421)	5.8 (196)	6.1 (225)	
Mother’s employment ^2^				0.740
Employed	87.0 (6436)	86.9 (3034)	87.1 (3402)	
Unemployed	13.0 (962)	13.1 (459)	12.9 (503)	
Quality of family relationship ^1^	8.47 ± 1.91	8.62 ± 1.79	8.34 ± 2.01	≤0.001
Family support ^1^	23.87 ± 6.45	24.24 ± 6.26	23.53 ± 6.60	≤0.001
Quality of life ^1^	36.43 ± 7.28	37.56 ± 7.58	35.49 ± 6.88	≤0.001
QoL below average ^2^	47.0 (2331)	37.9 (857)	* 54.6 (1474)	
QoL above average ^2^	53.0 (2631)	* 62.1 (1405)	45.4 (1226)	

^1^ Independent Sample *t*-test; ^2^ Chi-square. * Adjusted residuals > 1.96. Abbreviations: *M*, Mean; *SD*, standard deviation.

**Table 3:** Bivariate analysis between adolescents’ quality of life (QoL) and family environment.

	QoL Below Average	QoL Above Average	*p*
	% or *M* ± *SD*		
Age (years) ^1^	15.77 ± 1.83	15.31 ± 1.77	≤0.001
Sex ^2^			≤0.001
Male	36.8 (857)	* 53.4 (1405)	
Female	* 63.2 (1474)	46.6 (1226)	
School grade ^2^			≤0.001
8th grade	41.3 (963)	* 51.5 (1354)	
10th grade	32.1 (749)	29.1 (765)	
12th grade	* 26.6 (619)	19.5 (512)	
Region ^2^			≤0.001
North	34.1 (794)	* 39.7 (1045)	
Centre	19.2 (447)	18.5 (486)	
Lisbon and Tagus Valley	* 27.9 (650)	22.6 (595)	
Alentejo	9.0 (210)	9.8 (257)	
Algarve	9.9 (230)	9.4 (248)	
Family affluence ^1^	7.68 ± 2.38	8.17 ± 2.23	≤0.001
Low ^2^	* 30.6 (713)	21.9 (576)	
Medium ^2^	46.4 (1081)	49.3 (1298)	
High ^2^	23.0 (537)	* 28.8 (757)	
Communication with father ^2^			≤0.001
Easy	49.9 (1034)	* 74.4 (1819)	
Difficult	* 50.1 (1039)	25.6 (626)	
Communication with mother ^2^			≤0.001
Easy	72.6 (1645)	* 90.4 (2336)	
Difficult	* 27.4 (622)	9.6 (249)	
Living with both parents ^2^			≤0.001
No	* 31.5 (656)	24.9 (581)	
Yes	68.5 (1427)	* 75.1 (1757)	
Family meals ^2^			≤0.001
No	* 2.9 (67)	1.5 (40)	
Yes	97.1 (2264)	* 98.5 (2591)	
Parents help in decision making ^2^			≤0.001
No	31.3 (729)	7.1 (188)	
Yes	68.7 (1602)	92.9 (2443)	
Parents treat with fairness ^2^			≤0.001
No	25.5 (835)	6.9 (1287)	
Yes	74.5 (1349)	93.1 (1183)	
Pressure from parents to get good grades ^2^			≤0.001
No	38.2 (788)	* 52.1 (1334)	
Yes	* 61.8 (1196)	47.9 (1336)	
Father’s employment ^2^			≤0.001
Employed	92.1 (1922)	* 94.5 (2306)	
Unemployed	* 7.9 (164)	5.5 (133)	
Mother’s employment ^2^			≤0.05
Employed	85.5 (1911)	* 87.4 (2228)	
Unemployed	* 14.5 (325)	12.6 (321)	
Quality of family relationship ^1^	7.82 ± 2.12	9.05 ± 1.48	≤0.001
Family support ^1^	20.48 ± 7.40	25.56 ± 4.73	≤0.001

^1^ Independent Sample *t*-test; ^2^ Chi-square. * Adjusted residuals > 1.96. Abbreviations: *M*, Mean; *SD*, standard deviation.

**Table 4:** Linear regression between adolescents’ quality of life and family environment variables.

	Non-Standardized Coefficient		Standardized Coefficient	*t*
	B	Standard Error	*β*	
Family affluence	0.10	0.04	0.03 *	2.35
Communication with father	−1.34	0.22	−0.09 ***	−6.20
Communication with mother	−0.91	0.28	−0.09 ***	−3.24
Family meals	0.50	0.68	0.01	0.73
Parents help in decision making	−1.16	0.44	−0.06 **	−2.62
Parents treat with fairness	6.53	0.26	0.33 ***	25.25
Pressure from parents to get good grades	−1.83	0.19	−0.13 ***	−9.72
Quality of family relationships	0.57	0.06	0.15 ***	10.07
Family support	0.35	0.03	0.31 ***	12.60

The results were for age and sex. The variables were entered using the “enter” mode. * *p* ≤ 0.05; ** *p* ≤ 0.01; *** *p* ≤ 0.001.

## Data Availability

The data that support the findings of this study are available from the corresponding author upon reasonable request.

## References

[1] Fowler P.J., Henry D.B., Marcal K.E. (2015). Family and housing instability: Longitudinal impact on adolescent emotional and behavioral well-being. Soc. Sci. Res..

[2] Harcourt K.T., Adler-Baeder F., Erath S., Pettit G.S. (2015). Examining family structure and half-sibling influence on adolescent well-being. J. Fam. Issues.

[3] King V., Boyd L.M., Pragg B. (2018). Parent–adolescent closeness, family belonging, and adolescent well-being across family structures. J. Fam. Issues.

[4] Lee D., McLanahan S. (2015). Family structure transitions and child development: Instability, selection, and population heterogeneity. Am. Sociol. Rev..

[5] Thomas P.A., Liu H., Umberson D. (2017). Family relationships and well-being. Innov. Aging.

[6] Balistreri K.S., Alvira-Hammond M. (2016). Adverse childhood experiences, family functioning and adolescent health and emotional well-being. Public Health.

[7] Moreira J.F.G., Telzer E.H. (2015). Changes in family cohesion and links to depression during the college transition. J. Adolesc..

[8] Scott S.M., Wallander J.L., Elliott M.N., Grunbaum J.A., Chien A.T., Tortolero S., Cuccaro P., Schuster M.A. (2016). Do social resources protect against lower quality of life among diverse young adolescents?. J. Early Adolesc..

[9] Härkönen J., Bernardi F., Boertien D. (2017). Family dynamics and child outcomes: An overview of research and open questions. Eur. J. Popul..

[10] Davids E.L., Ryan J., Yassin Z., Hendrickse S., Roman N.V. (2016). Family structure and functioning: Influences on adolescents psychological needs, goals and aspirations in a South African setting. J. Psychol. Afr..

[11] Mitchell C., McLanahan S., Notterman D., Hobcraft J., Brooks-Gunn J., Garfinkel I. (2015). Family structure instability, genetic sensitivity, and child well-being. Am. J. Sociol..

[12] Perales F., Johnson S.E., Baxter J., Lawrence D., Zubrick S.R. (2017). Family structure and childhood mental disorders: New findings from Australia. Soc. Psychiatry Psychiatr. Epidemiol..

[13] Cavanagh S.E., Fomby P. (2019). Family instability in the lives of American children. Annu. Rev. Sociol..

[14] Dissing A.S., Dich N., Andersen A.M.N., Lund R., Rod N.H. (2017). Parental break-ups and stress: Roles of age & family structure in 44,509 pre-adolescent children. Eur. J. Public Health.

[15] Miranda M.C., Affuso G., Esposito C., Bacchini D. (2016). Parental acceptance–rejection and adolescent maladjustment: Mothers’ and fathers’ combined roles. J. Child Fam. Stud..

[16] Harrison M.E., Norris M.L., Obeid N., Fu M., Weinstangel H., Sampson M. (2015). Systematic review of the effects of family meal frequency on psychosocial outcomes in youth. Can. Fam. Physician.

[17] Mónaco E., Schoeps K., Montoya-Castilla I. (2019). Attachment styles and well-being in adolescents: How does emotional development affect this relationship?. Int. J. Environ. Res. Public Health.

[18] Mínguez A.M. (2020). Children’s relationships and happiness: The role of family, friends and the school in four European countries. J. Happiness Stud..

[19] Rattay P., von der Lippe E., Mauz E., Richter F., Hölling H., Lange C., Lampert T. (2018). Health and health risk behaviour of adolescents—Differences according to family structure. Results of the German KiGGS cohort study. PLoS ONE.

[20] Branje S. (2018). Development of parent–adolescent relationships: Conflict interactions as a mechanism of change. Child Dev. Perspect..

[21] Keijsers L. (2016). Parental monitoring and adolescent problem behaviors: How much do we really know?. Int. J. Behav. Dev..

[22] Marceau K., Ram N., Susman E.J. (2015). Development and lability in the parent–child relationship during adolescence: Associations with pubertal timing and tempo. J. Res. Adolesc..

[23] Bae D., Wickrama K.A. (2015). Family socioeconomic status and academic achievement among Korean adolescents: Linking mechanisms of family processes and adolescents’ time use. J. Early Adolesc..

[24] Nunes C., Hernando Á., Lemos I., Ayala-Nunes L., Oliva C.R., Coronado C.M. (2016). Quality of life of Portuguese and Spanish adolescents. A comparative study between natives and immigrants. Ciência. Saúde. Coletiva..

[25] Gaspar T., Matos M.G. (2015). As políticas amigas das pessoas e dos ambientes—Documentos orientadores: A qualidade de vida. Adolescentes—Navegação Segura por Águas Desconhecidas.

[26] Gaspar T., Gomez-Baya D., Trindade J.S., Guedes F.B., Cerqueira A., Matos M.G. (2021). Relationship Between Family Functioning, Parents’ Psychosocial Factors, and Children’s Well-Being. J. Fam. Issues.

[27] Lampropoulou A. (2018). Personality, school, and family: What is their role in adolescents’ subjective well-being. J. Adolesc..

[28] Wallander J.L., Schmitt M., Koot H.M. (2001). Quality of life measurement in children and adolescents: Issues, instruments, and applications. J. Clin. Psychol..

[29] González M., Gras M.E., Malo S., Navarro D., Casas F., Aligué M. (2015). Adolescents’ perspective on their participation in the family context and its relationship with their subjective well-being. Child Indic. Res..

[30] Bireda A.D., Pillay J. (2017). Perceived parent–child communication and well-being among Ethiopian adolescents. Int. J. Adolesc. Youth.

[31] Goldberg-Looney L.D., Sánchez-SanSegundo M., Ferrer-Cascales R., Smith E.R., Albaladejo-Blazquez N., Perrin P.B. (2015). Adolescent drinking in Spain: Family relationship quality, rules, communication, and behaviors. Child. Youth Serv. Rev..

[32] Ledwell M., King V. (2015). Bullying and internalizing problems: Gender differences and the buffering role of parental communication. J. Fam. Issues.

[33] Utter J., Denny S., Peiris-John R., Moselen E., Dyson B., Clark T. (2016). Family meals and adolescent emotional well-being: Findings from a national study. J. Nutr. Educ. Behav..

[34] Utter J., Larson N., Berge J.M., Eisenberg M.E., Fulkerson J.A., Neumark-Sztainer D. (2018). Family meals among parents: Associations with nutritional, social and emotional wellbeing. Prev. Med..

[35] Deb S., Strodl E., Sun H. (2015). Academic stress, parental pressure, anxiety and mental health among Indian high school students. Int. J. Psychol. Behav. Sci..

[36] Quach A.S., Epstein N.B., Riley P.J., Falconier M.K., Fang X. (2015). Effects of parental warmth and academic pressure on anxiety and depression symptoms in Chinese adolescents. J. Child Fam. Stud..

[37] Duncan G.J., Magnuson K., Votruba-Drzal E. (2014). Boosting family income to promote child development. Future Child..

[38] Gariepy G., Elgar F.J., Sentenac M., Barrington-Leigh C. (2017). Early-life family income and subjective well-being in adolescents. PLoS ONE.

[39] Otto C., Haller A.C., Klasen F., Hölling H., Bullinger M., Ravens-Sieberer U., BELLA Study Group (2017). Risk and protective factors of health-related quality of life in children and adolescents: Results of the longitudinal BELLA study. PLoS ONE.

[40] Crandall A., Magnusson B.M., Novilla M.L.B., Novilla L.K.B., Dyer W.J. (2017). Family financial stress and adolescent sexual risk-taking: The role of self-regulation. J. Youth Adolesc..

[41] Ponnet K., Van Leeuwen K., Wouters E., Mortelmans D. (2015). A family system approach to investigate family-based pathways between financial stress and adolescent problem behavior. J. Res. Adolesc..

[42] Jiménez-Iglesias A., Moreno C., Ramos P., Rivera F. (2014). What family dimensions are important for health-related quality of life in adolescence?. J. Youth Stud..

[43] Ma Z.W., Zeng W.N., Ye K.Y. (2015). Gender differences in Chinese adolescents’ subjective well-being: The mediating role of self-efficacy. Psychol. Rep..

[44] Inchley J.C., Currie D.B., Young T., Samdal O., Torsheim T., Augustson L., Mathison F., Aleman-Diaz A., Molcho M., Weber M. (2016). Growing up Unequal: Gender and Socioeconomic Differences in Young People’s Health and Well-Being: Health Behaviour in School-Aged Children (HBSC) Study: International Report from the 2013/2014 Survey.

[45] Matos M.G., Equipa Aventura Social (2018). A Saúde dos Adolescentes Portugueses Após a Recessão. Relatório do Estudo Health Behaviour in School Aged Children (HBSC) em 2018.

[46] Roberts C., Freeman J., Samdal O., Schnohr C., Looze M., Nic Gabhainn S., Iannotti I., Rasmussen M., Matos M.G. (2009). In the International HBSC study group The Health Behaviour in School-aged Children (HBSC) study: Methodological developments and current tensions. Int. J. Public Health.

[47] Boyce W., Torsheim T., Currie C., Zambon A. (2006). The family affluence scale as a measure of national wealth: Validation of an adolescent self-report measure. Soc. Indic. Res..

[48] Hartley J.E.K., Levin K., Currie C. (2015). A new version of the HBSC Family Affluence Scale—FAS III: Scottish Qualitative Findings from the International FAS Development Study. Child Indic. Res..

[49] Cantril H. (1965). The Pattern of Human Concerns.

[50] Jackson S., Bijstra J., Oostra L., Bosma H. (1998). Adolescents’ perceptions of communication with parents relative to specific aspects of relationships with parents and personal development. J. Adolesc..

[51] Tabak I., Mazur J., Granado Alcón M.D.C., Örkenyi Á., Zaborskis A., Aasvee K., Moreno C. (2012). Examining trends in parent-child communication in Europe over 12 years. J. Early Adolesc..

[52] Levin K.A., Dallago L., Currie C. (2012). The association between adolescent life satisfaction, family structure, family affluence and gender differences in parent–child communication. Soc. Indic. Res..

[53] Elgar F.J., Craig W., Trites S.J. (2013). Family dinners, communication, and mental health in Canadian adolescents. J. Adolesc. Health.

[54] Xiao Z., Li X., Stanton B. (2011). Perceptions of parent–adolescent communication within families: It is a matter of perspective. Psychol. Health Med..

[55] Inchley J., Currie D., Budisavljevic S., Torsheim T., Jåstad A., Cosma A., Kelly C., Már A. (2020). Spotlight on Adolescent Health and Well-Being. Findings from the 2017/2018 Health Behaviour in School-Aged Children (HBSC) Survey in Europe and Canada.

[56] Fulkerson J.A., Neumark-Sztainer D., Story M. (2006). Adolescent and parent views of family meals. J. Am. Diet. Assoc..

[57] Neumark-Sztainer D., Larson N.I., Fulkerson J.A., Eisenberg M.E., Story M. (2010). Family meals and adolescents: What have we learned from Project EAT (Eating Among Teens)?. Public Health Nutr..

[58] Gutman L.M., Schoon I., Sabates R. (2012). Uncertain aspirations for continuing in education: Antecedents and associated outcomes. Dev. Psychol..

[59] Marcenaro–Gutierrez O., Lopez–Agudo L.A., Ropero-García M.A. (2018). Gender differences in adolescents’ academic achievement. Young.

[60] Fortin N.M., Oreopoulos P., Phipps S. (2015). Leaving boys behind gender disparities in high academic achievement. J. Hum. Resour..

[61] Moore G.F., Cox R., Evans R.E., Hallingberg B., Hawkins J., Littlecott H.J., Long S.J., Murphy S. (2018). School, peer and family relationships and adolescent substance use, subjective wellbeing and mental health symptoms in Wales: A cross sectional study. Child Indic. Res..

[62] Duineveld J.J., Parker P.D., Ryan R.M., Ciarrochi J., Salmela-Aro K. (2017). The link between perceived maternal and paternal autonomy support and adolescent well-being across three major educational transitions. Dev. Psychol..

[63] Camacho I., Guedes F.B., Tomé G., Matos M.G. (2019). A família e os efeitos da guarda parental na relação e apoio familiar dos adolescentes. Rev. Psicol. D Criança E D Adolesc..

[64] Gaspar T., Matos M.G. (2017). Parenting Practices: Parent’s Perception of the Impact in Children Psychological Wellbeing. SM J. Fam. Med..

